# Factors influencing cervical cancer re-screening in a semi-rural health district of Cameroon: a cohort study

**DOI:** 10.1186/s12905-024-02917-3

**Published:** 2024-01-28

**Authors:** Sophie Evina Bolo, Bruno Kenfack, Ania Wisniak, Gilles Tankeu, Virginie Yakam, Alida Moukam, Jessica Sormani, Beat Stoll, Pierre Vassilakos, Patrick Petignat

**Affiliations:** 1 Department of Gynaecology and Obstetrics, Annex Regional Hospital of Dschang, Dschang, Cameroon; 2 Institute of Global Health, University of Geneva, Geneva, Switzerland; 3 Department of Obstetrics, Gynaecology and Maternal Health, University of Dschang, Dschang, Cameroon; 4 Division of Gynaecology, Department of Paediatrics, Gynaecology and Obstetrics, University Hospital of Geneva, Geneva, Switzerland; 5 Geneva Foundation for Medical Education and Research, Geneva, Switzerland; 6 Global Research Agency, Dschang, Cameroon; 7 HES-SO University of Applied Sciences and Arts Western, Delémont, Switzerland

**Keywords:** Screening, Human papilloma virus, Cervical cancer, Barriers, Sub-saharan Africa

## Abstract

**Background:**

Screening participation at recommended intervals is a crucial component of cervical cancer prevention effectiveness. However, little is known regarding the rate of re-screening in a Sub-Saharan context. This study aimed to estimate the re-screening rate of women in a semi-rural after an initial HPV-based screening and identify factors that influence adherence.

**Methods:**

This cohort study at the Annex Regional Hospital of Dschang enrolled women screened for cervical cancer over 5 years ago and due for re-screening. Women who initially tested HPV-positive (*n* = 132) and a random sample of HPV-negative women (*n* = 220) participated in a telephone survey between October 2021 and March 2022 to assess re-screening participation and reasons. Sociodemographic factors were collected, and associations with rescreening were evaluated.

**Results:**

A total of 352 participants aged under 50 years (mean age 37.4 years) were contacted, and 203 (58.0%) completed the survey. The proportion of women who complied with the screening recommendation was 34.0% (95% CI 27.5% − 40.5%), The weighted re-screening proportion was 28.4%. Age, marital status, education level, type of employment, and place of residence were not associated with the rate of re-screening. Main reported barriers to re-screening were lack of information (39.0%), forgetfulness (39.0%), and impression of being in good health (30.0%). Women who remembered the recommended screening interval were 2 to 3 times more likely to undergo re-screening (aOR (adjusted odds ratio) = 2.3 [1.2–4.4], *p* = 0.013). Human papilloma virus- positive status at the initial screening was also associated with the re-screening((aOR) (95% CI): 3.4 (1.8–6.5).

**Conclusion:**

Following an initial Human Papilloma Virus-based screening campaign in the West Region of Cameroon, one third of women adhered to re-screening within the recommended timeframe. Existing screening strategies would benefit from developing better information approaches to reinforce the importance of repeated cervical cancer screening.

## Background

Cervical cancer is the fourth most common cancer among women worldwide, with over 600,000 new cases in 2020 [1]. Nearly 90.0% of cervical cancer deaths occur in low-income countries, and the mortality rate is 18 times higher in low- and middle-income countries than in high-income countries [2, 3]. It is the leading cause of cancer death- related among women in sub-Saharan Africa, largely due to the lack of screening [2–4]. In Cameroon, it is the second most common cancer among women, after breast cancer [5, 6]. However, regular screening could reduce the risk of cervical cancer by 70.0% [7].

According to the World Health Organization’s (WHO) global strategy for the elimination of cervical cancer, 70.0% of the target population should be screened with an effective test such as the human papillomavirus (HPV) test, 90.0% of girls should be fully vaccinated against HPV by the age of 15, and 90.0% of women diagnosed with cervical disease should be treated by 2030 [4, 8]. This remains a challenge in low-income countries that face many obstacles that may hinder women’s access to cervical cancer screening services. Screening and treatment failures are related to difficulties such as lack of awareness among the target population, financial difficulties, and lack of adequate specialized health infrastructures [9, 10]. Long distances to travel to the few health facilities that offer screening, prohibitive transportation costs, negative attitudes towards patients, long waiting times, and lack of male support have been identified as major obstacles to accessing existing screening services [11–16]. For HPV-based screening, the WHO recommends regular screening at five-year intervals [8]. Adherence to regular screening is necessary for a program to be effective and should be monitored through longitudinal observation of screening participation. Only few studies have been conducted in low resource settings and available data evaluating one-year follow-up after HPV + testing support a low rate of adherence to cervical cancer re-screening (26.0%) for recommended screening [17].

In Cameroon, only 5.0% of women aged between 30 and 49 years have undergone screening in the past five years and, 5.0% of the female target population received their first dose of HPV vaccine [18]. In line with national guidelines recommending early and regular screening as well as treatment of precancerous and cancerous lesions [19], the Annex Regional Hospital of Dschang, Cameroon, in collaboration with the University Hospitals of Geneva, has established a cervical cancer screening unit with free clinical services since 2015 [20]. The aim of cervical cancer screening is not only getting women to initiate screening but also to encourage them to maintain regular use over time. To date, very little is known about participation rate of re-screening as well as factors that may help or hinder women’s participation to screening adherence over time. Yet, understanding determinants of re-screening appears essential for developing interventions to encourage women to be re-screened. The aim of our study was to determine the proportion of HPV-positive and HPV-negative women who attended cervical cancer rescreening within the recommended timeframe and factors influencing adherence and non-adherence to re-screening.

## Methods

### Study site

Our study was conducted in the Western Region of Cameroon, in Dschang, a university town, situated in the Menoua division. Dschang has an estimated population of around 176,940 inhabitants [21]. This is a follow-up study of a pilot study called the “3T approach” based on primary screening for HPV, implemented with the support of the Cameroonian Ministry of Health in 2015.

### Study type and design

This retrospective cohort study included women screened as part of the 3T-Approach (test, triage, treat) cervical cancer screening campaign organized at the Annex Regional Hospital of Dschang in collaboration with the University Hospitals of Geneva in 2015. Approximately 1012 women aged between 30 and 49 were included if they understood the study procedures, and voluntarily agreed to participate by signing an informed consent form. Women eligible for this study had to be under 44 years of age at the time of initial screening. Exclusion criteria were pregnancy, previous total hysterectomy, and inability to comply with the study protocol. Each included woman was primarily screened by an HPV test [22]. Women who tested negative for HPV received oral information from a qualified healthcare provider and a document reminding them of their next screening appointment in 5 years. Women who tested positive for HPV underwent a triage with visual inspection with acetic acid (VIA) and were treated free of charge if needed. Women having a positive HPV test underwent a follow-up screening test following the same procedure at 12 months, and, in case of negative results, received oral information from a qualified healthcare provider reminding them of their next screening appointment in 5 years. Adherence to the 1-year follow-up was of 80% [23]. For the present study, we considered for enrolment only women for whom a re-screening test was due and non-adherence was defined as not receiving at least two consecutive cervical cancer screening tests within a five-year schedule.

### Data collection

Sociodemographic data (age, education level, marital status, number of children, type of employment, place of residence) of participants were collected from the archives of the 2015 cohort. Re-screening data were collected over a 6-months period between October 2021 and March 2022. Participants were contacted by phone and interviews were conducted in French and/or English by a Cameroonian anthropologist (VY) and a physician (SE) based on a structured questionnaire developed by a team of Cameroonian and Swiss physicians and anthropologists experienced in cervical cancer screening in Cameroon. The questionnaires were pre-tested on 10 women and adapted accordingly. The final validated questionnaire was oriented along the following axes: (i) update of sociodemographic data and medical history of participants, as well as cervical cancer screening status, (ii) reasons for participation in re-screening, if any, (iii) reasons for non-participation in re-screening, (iv) experience of first screening and treatment, (v) support from the community, family or partner to attend screening, (vi) perception of cervical cancer and screening. Likert scale questions were used for sections (iv) and (v) of the survey. The participants who were not reachable during the first call were called back at least two more times at different times of the day and week. For those who remained unreachable, text messages containing information about the purpose of the call were sent. This method allowed us to maximize participation rate. The data collected during the calls were recorded using a paper form and then entered an electronic database for analysis using Secutrial® software. At the end of the study, the accuracy of all data was verified. Any inconsistencies were clarified by recalling the participant.

### Sample size

The study population consisted of 1012 women included in the 2015 cohort who were initially screened and/or treated. Among these, 728 women were eligible for our study, of whom 132 (18.1%) were positive for HPV. Assuming the proportion of women undergoing a new screening to be 20.0%, the inclusion of 246 women would have been necessary to obtain a precision of (+/− 5%) with a confidence level of 95.0%. However, considering a response rate to telephone questionnaires of 70.0%, based on our previous experiences with this study design, a total sample size of approximately 350 women was required. To achieve this sample size, we included all HPV-positive women (*n* = 132) and 220 randomly selected HPV negative women, for a total sample size of 352 women. Random selection of HPV-negative participants was done using the sample () function in R statistical software [24].

### Statistical analyses

The complete electronic dataset was analysed using SPSS 16 software [25]. Categorical variables were expressed as proportions, and 95% confidence intervals were estimated. Numeric variables were expressed as means with standard deviations or medians with interquartile ranges, as appropriate. Proportions between subgroups were compared using the Chi2 test or Fisher’s exact test, depending on the sample size, and means were compared using the t-test or Mann-Whitney test, depending on the sample distribution. Free responses to questions of the type “other: specify…” were grouped by categories of similar responses before being analysed. Associations between sociodemographic and clinical characteristics and cervical cancer re-screening were evaluated by simple and multivariable logistic regression. The weighted re-screening proportion was calculated by taking into account the proportions of HPV-positive and HPV-negative women at initial screening. All p-values less than 0.05 were considered statistically significant.

### Ethical considerations

This study is a continuation of the 3T-Approach 2015–2016 study approved by the Geneva Canton Ethics Council, Switzerland (CCER, N°2017 − 0110, and ceR-amendment n°3) and the National Ethics Committee for Human Health Research in Cameroon (N°2018/07/1083/CE/CNERSH/SP). Informed consent was obtained orally by telephone from each participant before the survey began, and all data collection forms were anonymized.

## Results

### Survey profile

**Fig. 1:**
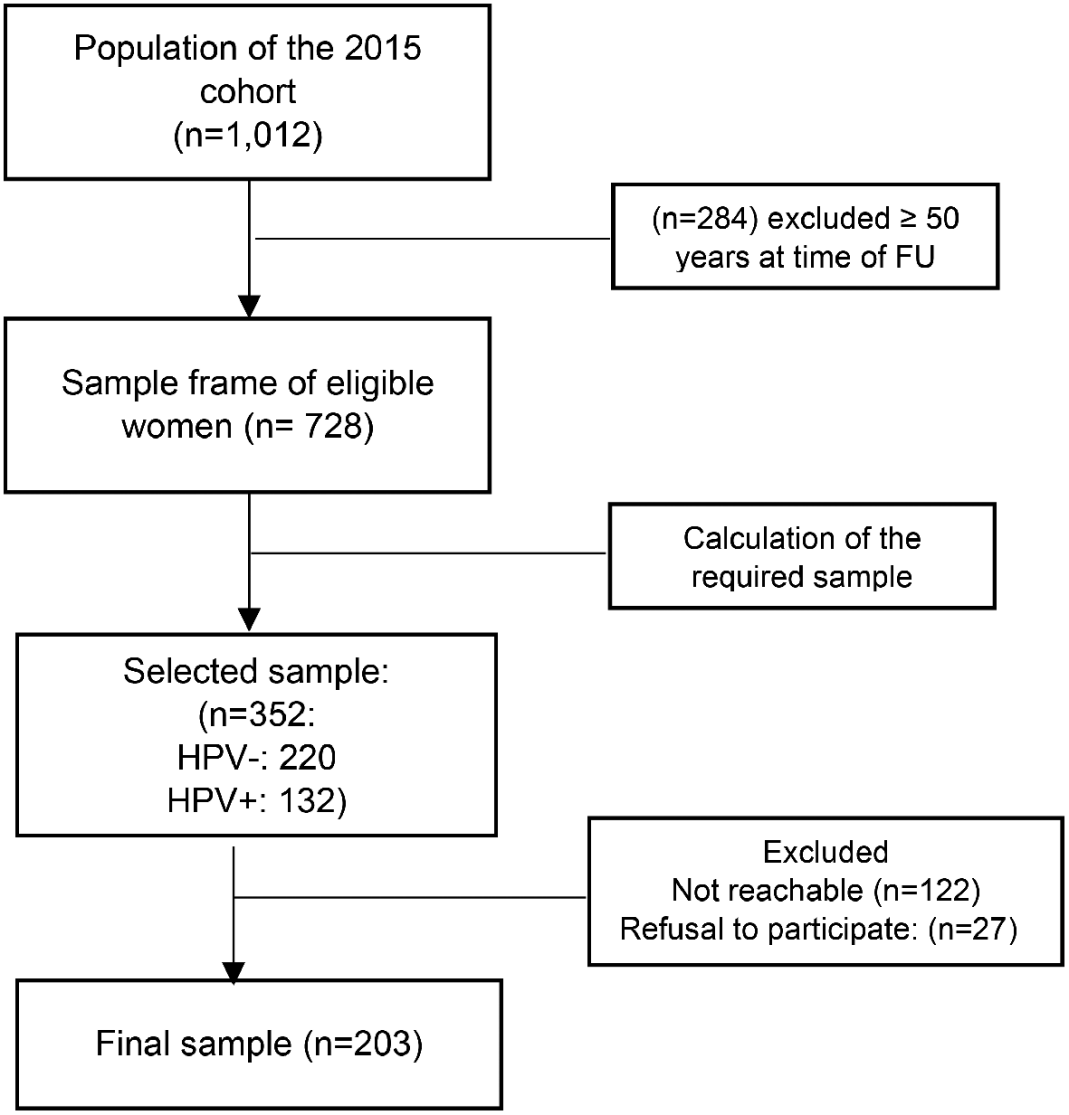
Participants flow chart. *Note* (n), number of patients, HPV: human papillomavirus. FU: follow up

A total of 203 female participants under 50 years old, including 88 (43.3%) positive for HPV and 115 (56.6%) negative, completed the questionnaire (participation rate of 58.0%). The average time between initial screening and inclusion in the study was 6.5 years, with a standard deviation (SD) of 0.1. One hundred and forty-nine participants were unable to complete our questionnaire: 27 participants refused to participate in the study, and 122 could not be reached (Fig. 1).

### Sociodemographic characteristics of study participants

The mean age of participants was 37.4 years. 86% were married or in a relationship, and 54.5% of participants had more than four children. Regarding education level, 57.6% had completed secondary school, 21.7% university, 15.3% primary school, 2.9% apprenticeship, and 1.0% had not completed any formal education. 75% of the participants lived in a semi-urban area. The most common type of employment or profession was salaried (50.4%); 30.7% were self-employed, 15.8% were housewives, and 2.9% were farmers (Table 1).

### Clinical characteristics

98% of the participants were non-smokers, and 84.7% had no known chronic diseases. Less than 1% were HIV-positive, 50.7% were HIV-negative, and 48.3% had not been tested for HIV for more than a year. Almost a third reported having a relative with cancer.

**Table 1:** Socio-demographic and clinical characteristics of participants

	n	(%)
Socio-demographic characteristics Age (average) 37.4 (+/−3.9)		
**Marital status**		
Single/widow/widower	16	(7.8)
Divorced/separated	11	(5.4)
Married/coupled	175	(86.2)
I did not mean	1	(0.4)
**Level of education**		
Primary	31	(15.2)
Learning/Secondary School	122	(60.1)
University	45	(22.1)
Not in school/Other	4	(1.9)
No response	1	(0.4)
**Area of residence**		
Rural	19	(9.3)
Semi-urban	153	(75)
Urban	31	(15.2)
Job/occupation* (if applicable)		
Employee	102	(50.2)
Farmer/Self-employed	60	(29.5)
Housewife/Other	41	(20.2)
**Number of children**		
≤ 4	92	(45.5)
> 4	110	(54.4)
Smoker* (*)		
Yes	4	(1.9)
No	199	(98)
Clinical features* (1)		
**Chronic illness**		
Present	31	(15.2)
Not present	172	(84.7)
**HPV status**		
HPV negative	115	(56.6)
HPV positive	88	(43.3)
**HIV infection**		
Yes	2	(0.9)
No	103	(50.7)
No screening for > 1 year	98	(48.2)
**Parent with cancer**		
Yes	60	(29.5)
No	139	(68.7)
Prefer not to answer	3	(1.4)
Missing	1	(0.4)

*Data updated during telephone calls in 2022

### Previous screening and/or treatment experience

98% of the participants reported being satisfied with the health care providers at their initial screening; among these, 97.5% reported being well-received, 81.3% were satisfied with the information received, and 70.7% reported feeling treated with respect.

### Screening practice

Of the 203 women who completed the questionnaire, 34.0% attended re-screening. with 40.6% attending at the recommended time (after at least 5 years). and 59.4% attending before the recommended date. The weighted re-screening proportion was 28.4%. 49% of re-screened women reported repeating screening because it was free; 26.1% because they were advised to do so by their relatives, and 18.8% because they had symptoms (pelvic pain, bleeding, etc.), 10.0% because it was the recommended date, 4.0% because they had been recalled, and 14.5% for other reasons (out of concern, during a routine visit, during a health campaign, by coincidence). Among those who attended re-screening, 79.7% had an HPV test, and 72.0% percent were re-screened at the annex regional hospital of Dschang.

### Obstacles to re-screening

The study found that 66% participants did not undergo re-screening since their participation in the 2015 campaign. Reported obstacles to rescreening included practical considerations, emotions related to screening, perception of one’s own health, and other reasons. In terms of practical considerations, 23.0% of participants stated that they did not repeat screening due to lack of time; 8.0% due to lack of money for transportation; 14.0% due to lack of available screening facilities; and 12.0% due to the long distance between their home and the hospital.

Regarding emotional reasons, 24.0% stated they did not repeat screening because they feared being diagnosed with cervical cancer, 5.0% because they feared the screening procedure would be painful, and 2.0% because they were embarrassed to have their private parts examined. Additionally, 30.0% did not undergo re-screening because they felt healthy, and 1.0% stated that their religious beliefs prevented them from being re-screened.

Participants were also asked to report any other reason that had prevented them from undergoing rescreening. In response to this question, 39.0% of participants said that lack of information was the problem, and the same proportion stated that they had forgotten that they needed to be screened again. Twenty-five per cent of participants mentioned other reasons such as neglect (14.0%), insecurity (2.0%), COVID-19 (1.0%), and others (8.0%).

**Fig. 2:**
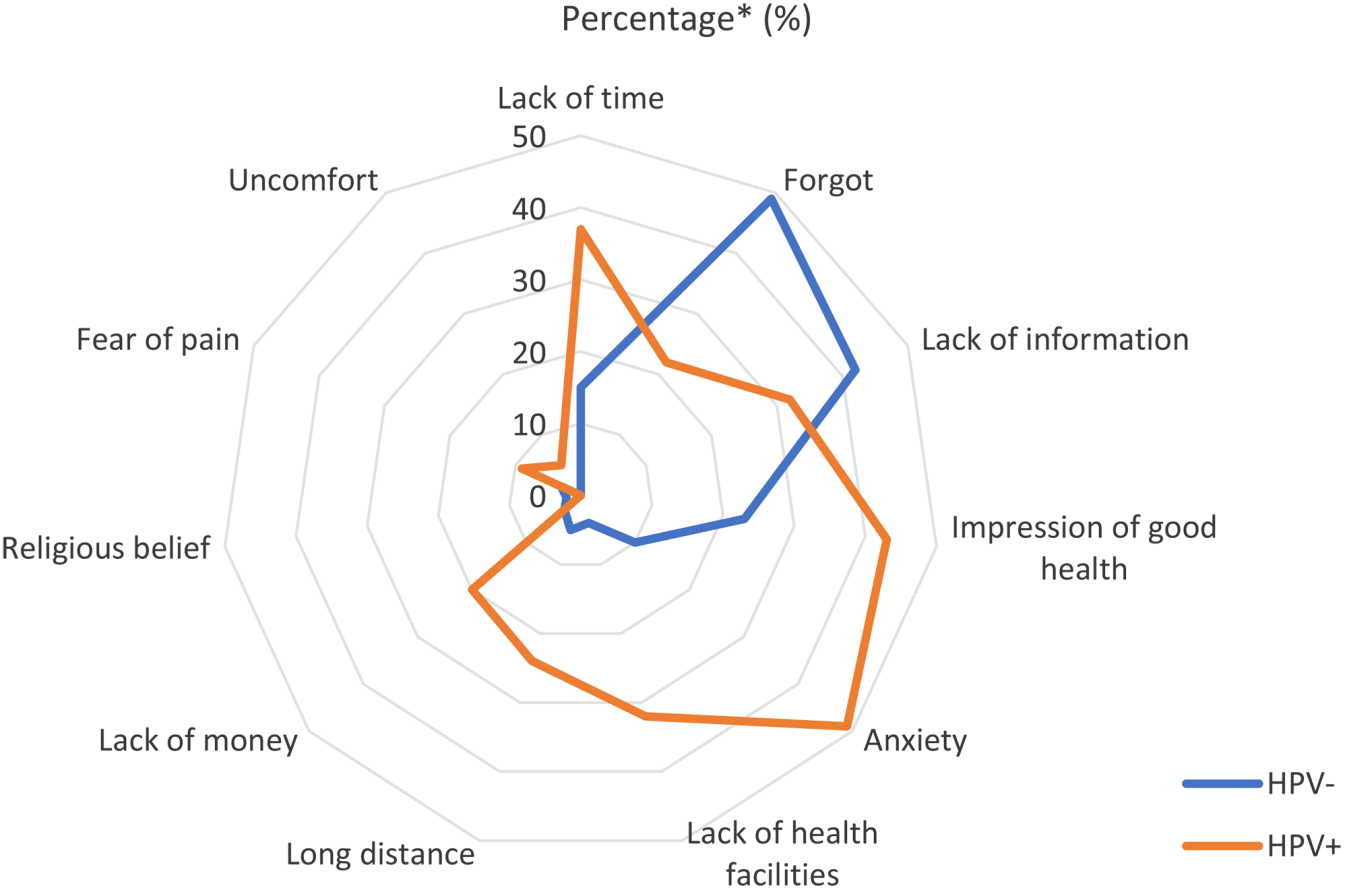
Barriers to the cervical cancer re-screening visit (HPV + and – stratified). *Percentage of participants who answered ‘agree’ and ‘strongly agree’ to questions related to barriers to CC re-screening

For HPV-positive participants specifically, the main obstacles to re-screening test were forgetfulness (49.0%), lack of information (42.0%), anxiety about repeating the test (49.0%), the impression of being healthy (43.0%), and lack of time (37.0%) (Fig. 2).

### Support from partner, family, or community

**Fig. 3:**
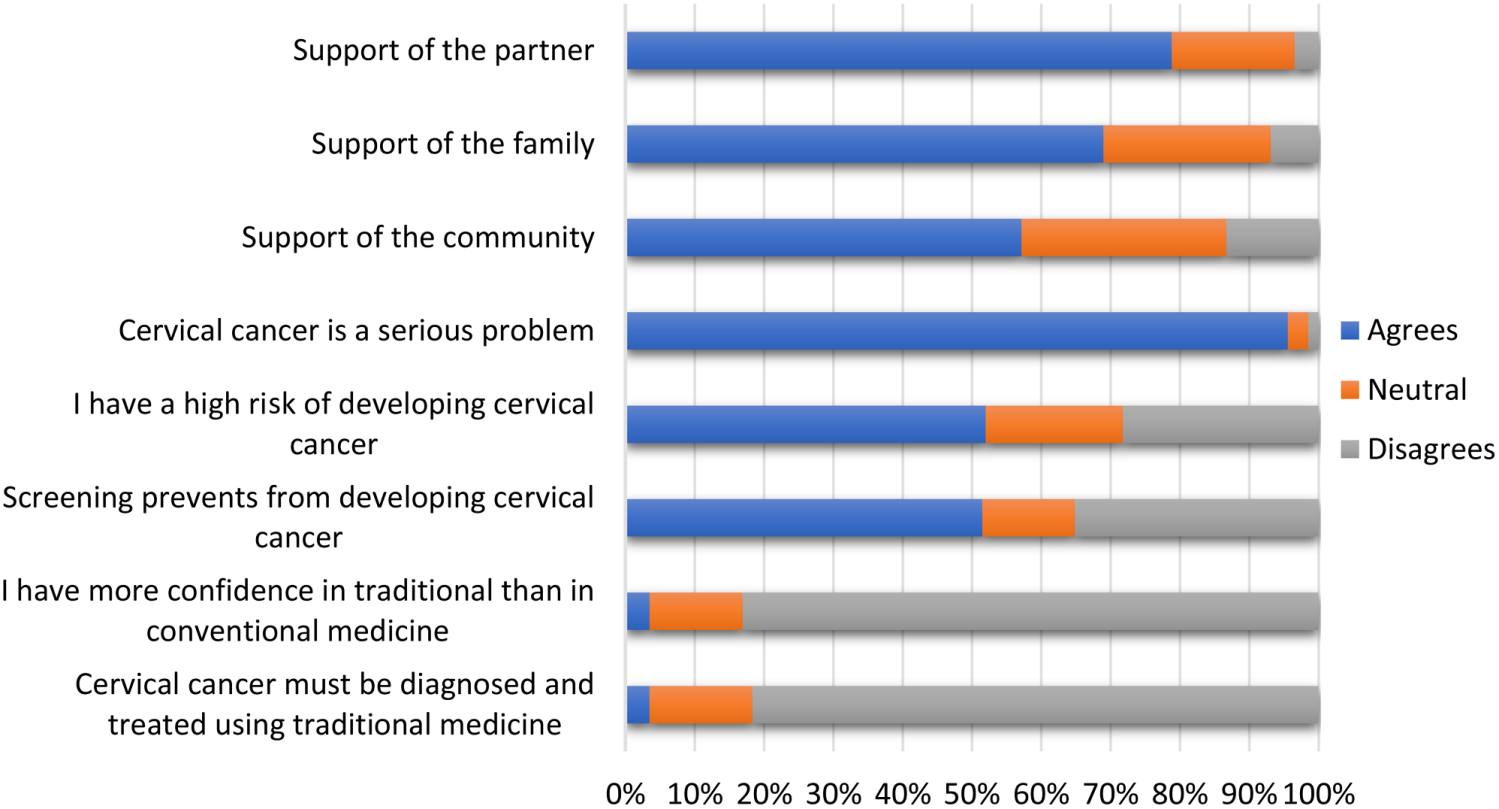
Beliefs and perceptions of cervical cancer screening

79% of participants reported receiving support from their spouse or partner; 69.0% from their family and 57.0% from the community (Fig. 3).

### Beliefs and perceptions of cervical cancer

More than 95.0% of participants believed that cervical cancer was a serious disease; 52.0% believed they were at high risk of cervical cancer, and about the same proportion (51.0%) believed that screening could prevent cervical cancer. Approximately 15.0% of women trusted traditional medicine more than conventional medicine; and 82.0% reported that cervical cancer should not be diagnosed and treated by traditional medicine (Fig. 3).

With respect to the knowledge of the recommended frequency of screening, the study found that 27.0% of participants knew that a woman should undergo cervical cancer screening every 5 years, which was the recommended frequency in the screening program. While 13.0% thought it should be done every 3 years and 28.0% every year, 31.0% said they did not know, and 1.0% believed it to be every 10 years. 81% of participants stated they would feel encouraged to undergo screening if it was recommended by the government; 78.0% if it was recommended by community outreach workers, and 87.0% if recommended by religious figures.

Regarding the cost of screening, 77.0% of participants stated they could undergo screening if the cost was between 5,000 and 10,000 FCFA (7.67 and 15.33 Euro), 38.0% if the cost was between 10,000 and 30,000 (15.33 and 46 Euro) and 32.0% between 30,000 and 50,000 FCFA (46 and 76,67 Euro).

### Associations between re-screening and participant characteristics

Associations between re-screening and family and medical history, as well as barriers to screening were examined. Only HPV status at initial screening and knowledge of recommended screening frequency were significantly associated with adherence to re-screening (*p* = 0.001 and *p* = 0.03, respectively). After adjusting for potential confounders, having a positive HPV status was associated with a 3 to 4 times higher risk of being screened again, compared to non-infected women (aOR = 3.4 [1.8–6.5], *p* < 0.001). Furthermore, women who remembered the recommended screening frequency were 2 to 3 times more likely to undergo new testing than those who did not remember (aOR = 2.3 [1.2–4.4], *p* = 0.013).

None of the evaluated sociodemographic characteristics were significantly associated with adherence to re-screening (Table 2).

**Table 2:** Factors associated with re-screening

	Test repeated		Test not repeated		cOR (95% CI)	p	aOR^a^ (95%CI)	p
	n	(%)	n	(%)				
**Total**	**69**	**(34.0)**	**134**	**(66.0)**				
**Age** ***(Mean (SD))***	*37.4*	*(3.7)*	*37.2*	*(4.0)*		0.6*		
[30–35] years	16	(23.2)	41	(30.6)	1		1	
[35–40] years	31	(44.9)	50	(37.3)	1.6 (0.8–3.3)	0.2	1.8 (0.8–3.9)	0.1
[40–50] years	22	(31.9)	43	(32.1)	1.3 (0.6–2.9)	0.4	1.6 (0.7–3.7)	0.2
**Civil status**								
Married/coupled	58	(84.1)	117	(87.3)	0.8 (0.3–1.7)	0.5	0.9 (0.4–2.2)	0.8
Single	11	(15.9)	17	(12.7)	1		1	
**Level of education**								
Primary + others	13	(19.1)	28	(20.9)	1		1	
Secondary school	42	(61.8)	75	(56.0)	1.3 (0.6–2.6)	0.6	1.1 (0.5–2.5)	0.7
University	13	(19.1)	31	(23.1)	0.9 (0.3–2.3)	0.8	0.9 (0.3–2.4)	0.8
**Area of residence**								
Rural	7	(10.1)	12	(9.0)	1		1	
Semi-urban	52	(75.4)	101	(75.4)	0.9 (0.3–2.4)	0.8	1.0 (0.4–2.8)	0.9
Urban	10	(14.5)	21	(15.7)	0.8 (0.2–2.7)	0.7	0.9 (0.2–3.1)	0.8
**Employment/profession**								
Housewife	18	(26.1)	23	(17.1)	1		1	
Self-employed	18	(26.1)	42	(31.3)	0.6 (0.2–1.2)	0.1	0.5 (0.2–1.1)	0.0
Employee	33	(47.8)	69	(51.5)	0.6 (0.3–1.3)	0.1	0.6 (0.2–1.2)	0.1
**Chronic illness**								
No	59	(85.5)	113	(84.3)	1		1	
Yes	10	(14.5)	21	(15.7)	0.9 (0.4–2.1)	0.8	1.0 (0.4–2.4)	0.9
**HPV status**								
HPV−	28	(40.6)	87	(64.9)	1			
HPV+	41	(59.4)	47	(35.1)	2.7 (1.5–4.9)	**0.001**		
**Parent with cancer**								
No	45	(65.2)	94	(70.7)	1		1	
Yes	24	(34.8)	39	(29.3)	1.3 (0.7–2.4)	0.4	1.2 (0.6–2.3)	0.5
**Frequency of screening**								
Knows	33	(47.8)	48	(35.8)	1.6 (0.9–3.0)	0.1	2.3 (1.2–4.4)	0.0
I don’t know.	36	(52.2)	86	(64.2)	1		1	
**Partner support**								
Present	52	(75.4)	108	(80.6)	0.7 (0.4–1.5)	0.3	0.9 (0.4–1.8)	0.7
Absent	17	(24.6)	26	(19.4)	1		1	

^a^Variables are adjusted to HPV status

## Discussion

In our study, we examined the participation of women who had their initial cervical cancer screening more than five years ago. Surprisingly, only 34.0% of these women had attended re-screening at the time of inclusion in our follow-up study. The weighted re-screening proportion was reduced to 28.4% when taking into account the ratio of positive to negative women at initial screening. Despite informing these women about the need for a repeat screening test five years later, our results reveal that we have not achieved optimal adherence to cervical cancer re-screening. This is particularly unexpected given the free screening program and the previous high level of adherence in this population [23]. To our knowledge, no other studies have investigated re-screening rates following a negative cervical cancer screening test in low- and middle-income countries. However, in a low-income population in Argentina, adherence of HPV-positive/cytology-negative women to follow-up testing at 12–18 months was 26.0% [17]. Low re-screening rates like these could hinder the long-term success of cervical cancer screening programs in resource-limited settings in reaching the 70% population coverage target set by the World Health Organization (WHO) has set a target of 70.0% population coverage [4, 8].

Our study also revealed that having a previous positive HPV test was associated with better adherence to re-screening (adjusted odds ratio: 3.4, 95% confidence interval: 1.8–6.5). This may reflect that women having a positive screening test may have a higher level of familiarity and commitment to the cervical cancer screening process, as they previously had more procedures and appointments than those with a negative HPV test. Furthermore, we identified several beliefs and perceptions about cervical cancer which could play a role in adherence to re-screening. Only a half of the participants believed that screening could prevent cervical cancer, and rough equal proportion believed that they were at high risk for the disease. This suggests that improving knowledge about the effectiveness of screening and raising awareness about cervical cancer risk could encourage more women to undergo re-screening. In terms of obstacles to re-screening, our study indicates that the main reasons were lack of information, forgetfulness, and a perception of good health. Our findings also showed that women who knew the recommended screening frequency were 2 to 3 times more likely to undergo re-screening within the recommended timeframe. Lack of information about cervical cancer has been a common issue in studies conducted in similar contexts [11, 26–29].

Therefore, it is essential for the Ministry of Health to prioritize communication and the dissemination of clear, appropriate information on best screening practices. Tailored information campaigns should be developed to reach the target population, both in public spaces (markets, streets, schools, universities, women’s associations, etc.) and in healthcare facilities, such as gynecology/obstetrics departments and pediatric vaccination clinics, where cervical cancer screening could be integrated into other healthcare services. Additionally, involving healthcare personnel in promoting cervical cancer screening has been effective in similar contexts [27, 30]. It would therefore be relevant to involve health personnel in promotion of cervical cancer screening among women attending health care facilities for other reasons. Implementing communication and information strategies, such as SMS recall systems, like the successful “call and recall” system used in the United Kingdom in 1988, could also boost cervical cancer re-screening rates in line with WHO recommendations [31]. Among HPV-positive participants, anxiety related to the possibility of having cancer was a major concern, outweighing other reasons. Several studies have shown that HPV-positive women, whether with abnormal or normal cytology results, experience higher short-term anxiety than those with normal results [32, 33]. Thus, it is crucial to train healthcare providers to provide reassurance to HPV-positive patients.

Interestingly, more than half of our study population (57.0%) reported having community support when it came to undergoing cervical cancer screening. This is an encouraging finding and warrants further exploration for promotional activities in similar settings, as community support has been shown to facilitate screening uptake [30]. Notably, we did not observe any significant associations between sociodemographic factors and cervical cancer re-screening in our study population. In other African studies, unemployed women were less likely to be screened than employed women [34, 35]. However, during our study, the provision of free screening removed financial barriers for our participants.Cultural barriers did not seem to be a significant issue in our population, as the majority (82.0%) did not believe that traditional medicine should be used for diagnosing and managing cervical cancer. Only a small percentage (1.0%) perceived religious beliefs as an obstacle to screening, although this could be influenced by the fact that this population had already been screened once. Additionally, a large majority (86.0%) stated that they would undergo screening if recommended by the government. Furthermore, most women (76.0%) stated that they could afford screening for a fee ranging from 5,000 to 10,000 CFA francs (approximately 8–16 Euros). These findings should be considered in the development of a national strategy for cervical cancer prevention in Cameroon, with an emphasis on universal health coverage, given the risk of inequitable access to screening. Our study indicates that nearly a quarter of women would not have access to screening if it were to be paid for.

### Limitations and strengths

To the best of our knowledge, this is the first report of re-screening rates in an HPV-based cervical cancer study in Sub-Saharan Africa. However, we must interpret our study findings with some important considerations. Firstly, our study focused on a specific group of women predominantly residing in a semi-urban area who accessed screening and pre-cancerous cervical lesion treatment at a local district hospital, with screening and transport costs covered. This unique setting may have obscured the presence of financial barriers to re-screening that could be more common in other situations.

Second, challenges in reaching participants from the original study cohort led to a relatively low participation rate. Consequently, adherence to re-screening may have been overestimated due to a participation bias, as women accepting to take part in the study were potentially more likely to be those having adhered to re-screening recommendations.

## Conclusion

Our study reveals that only one-third of participants underwent re-screening within the recommended timeframe. The primary barriers reported included a lack of information, forgetfulness, and the perception of being in good health. Nevertheless, early, and timely detection of precancerous lesions is critical to preventing long-term morbidity and mortality associated with cervical cancer. To fully realize the benefits of screening, it is essential to explore new approaches for educating women about the importance of regular cervical cancer screening. Further research should be conducted to assess strategies aimed at improving adherence to re-screening.

## Data Availability

The datasets used and/or analysed during this study are available from the corresponding author upon reasonable request.

## **Abbreviations**

CC: cervical cancer
ARHD: Annex Regional Hospital of Dschang
HPV: Human Papilloma Virus
HPV+: Human Papilloma Virus Positive
HPV−: Human Papilloma Virus Negative
VIA: Visual Inspection with Acetic Acid
WHO: World Health Organisation
